# An Enhanced VLC Channel Model for Underground Mining Environments Considering a 3D Dust Particle Distribution Model

**DOI:** 10.3390/s22072483

**Published:** 2022-03-24

**Authors:** Pablo Palacios Játiva, Cesar A. Azurdia-Meza, Iván Sánchez, David Zabala-Blanco, Ali Dehghan Firoozabadi, Ismael Soto, Fabian Seguel

**Affiliations:** 1 Department of Electrical Engineering, Universidad de Chile, Santiago 8370451, Chile; pablo.palacios@ug.uchile.cl; 2 Escuela de Informática y Telecomunicaciones, Universidad Diego Portales, Santiago 8370190, Chile; 3 UDLA Telecommunications Engineering Degree FICA, Universidad de Las Américas, Quito 170503, Ecuador; 4 Department of Computing and Industries, Universidad Católica del Maule, Talca 3466706, Chile; dzabala@ucm.cl; 5 Department of Electricity, Universidad Tecnológica Metropolitana, Av. Jose Pedro Alessandri 1242, Santiago 7800002, Chile; adehghanfirouzabadi@utem.cl; 6 Department of Electrical Engineering, Universidad de Santiago de Chile, Santiago 9170124, Chile; ismael.soto@usach.cl (I.S.); fabian.seguelg@usach.cl (F.S.)

**Keywords:** dust particle distribution modeling, scattering, underground mining visible light communication (UM-VLC), VLC channel modeling

## Abstract

Underground Mining (UM) is a hostile industry that generally requires a wireless communication system as a cross-cutting axis for its optimal operation. Therefore, in the last five years, it has been shown that, in addition to radio-frequency-based communication links, wireless optical communications, such as Visible Light Communication (VLC), can be applied to UM environments. The application of VLC systems in underground mines, known as UM-VLC, must take into account the unique physical features of underground mines. Among the physical phenomena found in underground mines, the most important ones are the positioning of optical transmitters and receivers, irregular walls, shadowing, and a typical phenomenon found in tunnels known as scattering, which is caused by the atmosphere and dust particles. Consequently, it is necessary to use proper dust particle distribution models consistent with these scenarios to describe the scattering phenomenon in a coherent way in order to design realistic UM-VLC systems with better performance. Therefore, in this article, we present an in-depth study of the interaction of optical links with dust particles suspended in the UM environment and the atmosphere. In addition, we analytically derived a hemispherical 3D dust particle distribution model, along with its main statistical parameters. This analysis allows to develop a more realistic scattering channel component and presents an enhanced UM-VLC channel model. The performance of the proposed UM-VLC system is evaluated using computational numerical simulations following the IEEE 802.1.5.7 standard in terms of Channel Impulse Response (CIR), received power, Signal-to-Noise-Ratio (SNR), Root Mean Square (RMS) delay spread, and Bit Error Rate (BER). The results demonstrate that the hemispherical dust particle distribution model is more accurate and realistic in terms of the metrics evaluated compared to other models found in the literature. Furthermore, the performance of the UM-VLC system is negatively affected when the number of dust particles suspended in the environment increases.

## 1. Introduction

Despite being an industry that generates a large part of Gross Domestic Product (GDP) for many countries worldwide, the Underground Mining (UM) work environment and its physical characteristics are considered dangerous and unsafe [1,2]. External factors generated by the regular operation of the mine, such as toxic components, heavy metals, and dust particles make work in UM tunnels hostile. Therefore, to generate greater control of mining activity, it is necessary to establish reliable and stable communication systems in order to manage day-to-day communication, along with emergencies that may occur in this environment. However, the physical structure of UM tunnels is a challenge for the design and implementation of systems that allow stable and effective communication [3].

Among the current communication systems applied to UM, wired communication systems (copper, coaxial, or fiber) are susceptible to damage by the working environment and are not reliable in UM tunnels. In terms of wireless communication systems, radio communication systems exhibit high signal attenuation due to electromagnetic interference in mining tunnels [4]. It is also important to mention that these environments need good lighting that meets international standards for safe work [5]. These challenges and disadvantages of the current technologies implemented in UM are presented as opportunities to research and develop complementary communication systems that guarantee efficient communication and optimize the lighting in UM environments simultaneously. Therefore, to address these communication and lighting problems in a single system, the concept of Visible Light Communication (VLC) has been introduced for UM communication [6,7].

There are several benefits of applying VLC-based technologies in UM, such as unlicensed spectrum usage capacity ranging from 400 THz to 800 THz, cost versatility for VLC system components, environmental friendliness, and immunity to electromagnetic interference [8]. In terms of the VLC system and its elements, the basic components of a VLC system for any type of environment are (a) Light-emitting diodes (LED), which are made up of cold light, and form lamps with a long useful life. The LED lamps can be located on the ceiling or walls of the UM tunnels. (b) Photo-Diodes (PDs) that could be installed in machinery, devices that need to be controlled, or the helmet of the mining worker. This scenario creates a VLC link between devices or workers and the mining infrastructure, or also between devices, which favors the design of multiple applications [9]. (c) The channel model, which is an important step for an efficient, reliable, and robust VLC system design [10].

According to the specialized literature, with the process of modeling the communication channel realistically and efficiently, the overall performance of the communication system can be improved [11]. Furthermore, adequate modeling ensures a correct approach and contextualization of the problems that may arise in communication systems [7]. Specifically for VLC systems applied to underground mines, a VLC channel model that considers the physical characteristics that affect UM tunnels was proposed [12]. Here, the random orientation of optical transmitters and receivers, which impact directly the Line-of-Sight (LoS) and Non-Line-of-Sight (NLoS) components, were considered in the mathematical model. In addition, the characterization of non-flat walls in tunnels and their reflection effects on the optical signal were considered. Finally, a shadowing pattern that considers the entry of objects into the UM tunnel and a scattering model based on a random distribution of dust particles around the PD on a 2D disk was considered.

In indoor environments such as offices, homes, or hospitals, the effect that suspended dust particles can generate on the optical links is neglected. Consequently, VLC systems applied to these scenarios do not compromise their performance due to the scattering phenomenon. In contrast, in UM environments, drilling and rock excavation work produce a large amount of suspended dust. Therefore, it is a major challenge to efficiently model the scattering effect and include it in the overall UM-VLC channel model. It should be noted that the scatterer distribution model used in [12] is easy to develop and implement. However, it was presented as an initial basic model that must be improved to make it more realistic. Since scatterers are considered multi-path components in the UM-VLC channel model, the received multi-path signal plays an important role in VLC system performance analysis. Therefore, the development of more realistic scatterer distribution models around the behavior of the dust particles in the tunnel must be analyzed and studied. This will allow us to make the channel model presented in [12] more accurate and realistic in its representation of the UM tunnel.

In the following subsections, we first discuss various models of general scatterer distributions based on stochastic geometry proposed in the state of the art. Then, studies of the effects and influence of dust particles on the optical signal applied to several VLC systems are presented.

### 1.1. Works Related to General Scatterer Distribution Models

Several works related to scattering distribution models applied to general communication schemes are presented in [13,14,15,16,17]. In [13,14], a uniform distribution model that locates the scatterers in a 2D disk region centered on the optical receiver is presented. For the system modeling, the authors consider a mobile station as a receiver. Although the focus of the model presented is not VLC systems, this proposal could be applied to any type of technology based on wireless communication, which includes dust particles that generate scattering. In [15], the authors present a statistical analysis in terms of arrival time and arrival direction of a geometric channel model that considers a mobile transmitting station and scatterers distributed in a hemispheric area around a receiving base station. The results show that this scatterer distribution can be implemented in any multi-path wireless communication system. The manuscript presented in [16] introduces to the state of the art a Gaussian model of scatterer distribution. The authors mention that this model is applicable to multi-path wireless communication systems due to its spatial and temporal properties. The demonstrated statistical results are presented in terms of arrival angle and arrival time. Finally, the work in [17] proposes a general channel model for any communication system that includes scattering particles. This three-dimensional model is based on stochastic geometry, where a Gaussian distribution of scatterers located around an arbitrary point within a spheroid is assumed. Furthermore, the transmitter and receiver are considered to be located at the focal points of this spheroid. Statistics in terms of the arrival angle and the arrival time of the model are obtained. Being a generalized channel model, this proposal could be extrapolated to wireless communication systems in indoor environments, in order to evaluate their performance.

As we can see in the analyzed literature, a large part of the proposed scatterer distribution models can be implemented in any type of multi-path wireless communication system, both indoors and outdoors. This advantage implies that they can also be extrapolated to VLC systems applied to underground mines.

### 1.2. Scatterer Distribution Models Applied to Optical Communication Systems

After an in-depth literature review, it can be seen that there are some works that present scatterer distribution models applied to optical communication systems [12,18,19,20,21,22,23]. In [18,19], a spread spectrum wireless dispersion model based on an NLoS optical component is presented. The paradigm of these works is based on the fact that air molecules and suspended aerosols build NLoS optical communication links caused by scattering when using carriers in the infrared light band. References [20,21] are the pioneering research by including and modeling the effect of scattering in indoor VLC environments. To model scattering, several particles are randomly placed in a 2D ellipse or ring shape. Finally, the complete VLC channel model is derived by the arithmetic add of the LoS channel component and the channel components produced by scattering. In [22] the authors propose a proposes a 3D Regular-Shaped Geometry-Based Stochastic Model (RS-GBSM) for vehicular VLC Multiple-Input Single-Output (MISO) channels. The distribution proposed combines a two-sphere model and an elliptic-cylinder model. The results of this work demonstrate the relationship between the communication range and the elevation angle in the proposed model on the received optical power. The authors in [12] present a novel channel model that includes in its analysis influential factors in the UM-VLC system. Among the characteristics considered are the random orientation of the LED and PD, irregular walls that produce reflections with random directions, shadowing caused by machinery and scattering caused by suspended dust particles. Although the VLC channel model presented considers important components of the UM environment, the scatterer distribution model implemented is simplistic. This model is based on the assumption that the dust particles are distributed within a two-dimensional disk-shaped area. Therefore, this scheme should be improved to have a more accurate and realistic UM-VLC channel model. Finally, the most recent work on the study of dust particles and their influence on VLC systems applied to underground coal mines is presented in [24]. The focus of this manuscript is based on the influence of coal dust particles and the scattering model. Furthermore, the impact of coal dust particles on visibility and attenuation is analyzed for visible light transmission. Although the work analyzes the effect of scatterers (dust particles) on the performance of the VLC system, the article does not present a specific mathematical and analytical model of scatterer distribution for the tunnel environment. A VLC channel component produced by the interaction of light with scatterers is also not specified.

As observed in the state of the art analysis of scatterer models applied to optical communications systems, the consideration and implementation of dust particles distributions that closely emulate their behavior in UM environments and their effect on VLC systems. Neither an exhaustive analysis on the interaction of light bonds with the atmosphere and dust particles in UM tunnels has been made. Based on these premises and opportunities to improve the existing state-of-the-art UM-VLC channel models, the main contributions of this work are presented below:

1. An in-depth analysis of the absorption and scattering parameters in the UM environment and the interaction of the incident light in the VLC system with the dust particles and the atmosphere of the UM environment was performed.
2. A novel 3D dust particle distribution model based on a hemispherical region, along with its mathematical parameters and its statistics based on the joint distribution of the arrival time and the arrival angle of the scattering down-link were developed and analytically proposed.
3. A more accurate and realistic UM-VLC channel component produced by dust particles (scattering), which enhances the general UM-VLC channel model, was derived and presented.
4. The performance of the proposed enhanced UM-VLC channel model was examined, evaluated, and compared with a state-of-the-art UM-VLC channel model in terms of CIR, received power, SNR, RMS delay spread, and BER.

The remainder of this paper is organized as follows. In Section 2, a brief contextualization of the UM-VLC channel model used is presented. In Section 3, the analysis of the scattering phenomena in the UM scenario and the 3D hemispheric dust particles distribution model are presented. In Section 4, the most relevant evaluation metrics applied to the UM-VLC system through numerical results are evaluated and discussed. Finally, relevant conclusions are presented in Section 5.

## 2. Underground Mining VLC System Model

**Figure 1:**
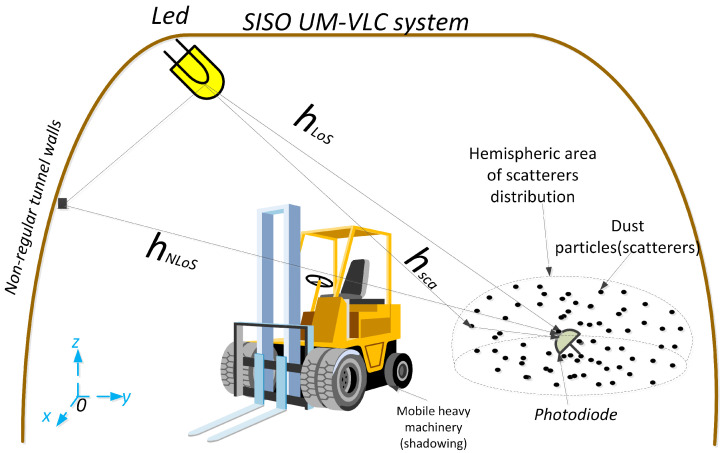
Geometry of the channel components that make up the SISO UM-VLC system.

In this article, we consider a Single-Input Single-Output (SISO) UM-VLC down-link system, as shown in Figure 1. This scenario includes the following characteristics, which are common in UM environments: (a) a randomly positioned and oriented LED between the ceiling and wall of the mining tunnel, which serves as an optical transmitter, (b) a PD located and randomly oriented at a height determined by the common position of devices on the tunnel, which serves as an optical receiver. (c) Irregular walls, which generate random reflections of the optical signal that directly affect the non-LoS component of the UM-VLC channel (d) Random shadowing produced by heavy machinery, which can partially or totally block the LoS or non-LoS channel components, (e) scattering produced by dust particles, which are generated by the work environment of a mining tunnel [12]. Under this context, this article focuses its study on the interaction of dust particles and the atmosphere with the light beam in the UM-VLC system, its basic absorption and scattering parameters, and the analysis of the dust particles distribution that best adapts to the UM environment, in order to obtain a better UM-VLC channel model component produced by dust particles (scattering) in the scenario.

In this work, the LED transmission is based on the PHY-I mode together with the On-Off Keying (OOK) modulation, which are described in the IEEE 802.1.5.7 standard. On the other hand, the PD reception employs asynchronous symbol-by-symbol detection [25,26]. Under this context, the received signal, *r*, is expressed as [25,26]

(1) $$r=h_{m}⊗s+n_{m},$$

where *s* denotes the transmitted symbol, $h_{m}$ represents the instantaneous UM-VLC channel-gain coefficient, $n_{m}$ stands for the noise affecting the UM-VLC system, and ⊗ is the convolution operator. Where $n_{m}$ = $n_{s}$ + $n_{t}$, $n_{s}$ ∼N(0, $\sigma_{s}^{2}$), and $n_{t}$ ∼N(0, $\sigma_{t}^{2}$), being $\sigma_{s}^{2}$ and $\sigma_{t}^{2}$ the variances of the shot noise, $n_{s}$, and thermal noise, $n_{t}$, respectively [25].

Based on the phenomena and features previously mentioned, the characterization of $h_{m}$ can be written as [12]

(2) $$h_{m}=h_{LoS}+h_{NLoS}^{\left(1\right)}+h_{sca},$$

where $h_{LoS}$, $h_{NLoS}^{\left(1\right)}$, and $h_{sca}$ represent the channel gains of the LoS link, the NLoS link, which represents the light reflections on the tunnel walls, and a component produced by the scattering, respectively. We must consider that the shadowing phenomenon harmfully affects the $h_{LoS}$ and $h_{NLoS}^{\left(1\right)}$ components, which is included in them as a coefficient that takes values between 0 and 1 to indicate a partial or total blocking of the link. The analytical and mathematical derivation together with the justification of the variables included in the components of the UM-VLC channel model are presented in detail for in-depth verification in [12].

In the next section we will analyze in depth the scattering phenomenon that occurs in the UM scenario and we will present the proposed 3D dust particle distribution model, which is based on a hemispheric area around the user’s helmet.

## 3. Analysis of the Scattering Phenomenon in Underground Mines and Distribution of Dust Particles

In UM environments, the work of people and machinery to drill, crush and fly rock generates large amounts of suspended dust particles. Therefore, it is necessary to first analyze the interaction of the light beam with the dust particles, then generate a dust particle distribution model to finally model the scattering effect and introduce it as a component in the complete UM-VLC channel model.

### 3.1. Atmospheric and Dust Particles Scattering and Absorption Parameters in UM

The distribution and concentration of dust particles suspended in mining tunnels have a strong impact on the propagation of the optical signal and, consequently, on the performance of the VLC system. In general, dust particles can generate two phenomena: (a) absorption of light when dust particles combine with water vapors and the light beam hits them, (b) scattering of light when dust particles consist only of coal or other organic matter and the light beam hits them. Both events can generate severe attenuation of the optical link. Therefore, the relationship between the intensity of the incident light and the intensity of the transmitted light, which can be considered as the intensity attenuation, can be expressed as follows

(3) $$I_{R}=I_{T}\left(\alpha_{T},\beta_{T}\right)\exp\left(-\frac{1.5CLK_{e}}{d}\right),$$

where *C* is the number of suspended particles in the unit volume, also called dust particle concentration, $\alpha_{T}$ and $\beta_{T}$ represent the rotation and tilt angles of the LED respectively, *L* is the light path, *d* denotes the Euclidean distance between the LED and PD, and $K_{e}$ is the light attenuation coefficient, which is represented with the expression $K_{e}=k_{sca}+k_{abs}$, being $k_{sca}$ the scattering coefficient and $k_{abs}$ the absorption coefficient. Furthermore, *C* is a dynamic parameter that can be modeled as an exponential function in terms of the visibility of the UM environment (*V*) under the expression $C=4050V^{-1.016}$.

If we consider the basic theory of light atmospheric transmission, $k_{sca}$ and $k_{abs}$ allow us to determine the average distance that a photon travels before being scattered or absorbed, respectively. This consideration can be applied to mining tunnels since dust particles can be assumed as aerosols of various sizes. Under this context, the effect of scattering and absorption produced by the interaction of light with dust particles can be modeled under the paradigms of the Rayleigh and Mie theory, respectively [18,19]. Therefore, $k_{sca}$ and $k_{abs}$ are the sum of molecule and aerosol scattering and absorption coefficients, represented as

(4) $$k_{sca}=k_{sca}^{Ray}+k_{sca}^{Mie},$$

(5) $$k_{abs}=k_{abs}^{Ray}+k_{abs}^{Mie},$$

where $k_{sca}^{Ray}$ and $k_{sca}^{Mie}$ represent the Rayleigh and Mie scattering coefficients respectively, and $k_{abs}^{Ray}$ and $k_{abs}^{Mie}$ are the Rayleigh and Mie absorption coefficients respectively. Since the composition of air molecules and dust particles is relatively constant in low-altitude UM areas, $k_{sca}^{Ray}$ is given by

(6) $$k_{sca}^{Ray}=\frac{{\left(m_{s}^{2}-1\right)}^{2}\left(6+3\delta \right)24\pi^{3}}{{\left(m_{s}^{2}+2\right)}^{2}\left(6-7\delta \right)\lambda^{4}N_{s}},$$

where $N_{s}$ is the molecular number density for air in UM, λ is the wavelength of the transmitted light beam, δ is the depolarization factor, and $m_{s}$ is the refractive index, which at the level of the mining tunnels is expressed as

(7) $$\left(m_{s}^{2}-1\right)\times 10^{8}=8060.51+\frac{2,480,990}{132.274-\lambda^{-2}}+\frac{17,455.7}{39.32957-\lambda^{-2}}.$$

On the other hand, $k_{abs}^{Ray}$ is a combination of different types of light absorption produced by gases such as $O_{2}$, $O_{3}$, ${\mathrm{CO}}_{2}$, among others, which are typical in UM environments. The absorption of light produced by these gases at different wavelengths can be represented through the Atmospheric Radiation Transport Model (MODTRAN), as shown in [27].

In terms of $k_{sca}^{Mie}$ and $k_{abs}^{Mie}$, these coefficients can be modeled based on the Mie theory through a dust particle size distribution with arbitrary form $n(r_{d})$, which is expressed as

(8) $$k_{sca}^{Mie}=\int_{0}^{\infty }\pi r_{d}^{2}Q_{sca}\left(r_{d},\lambda ,m\right)n\left(r_{d}\right)dr_{d},$$

(9) $$k_{abs}^{Mie}=\int_{0}^{\infty }\pi r_{d}^{2}Q_{abs}\left(r_{d},\lambda ,m\right)n\left(r_{d}\right)dr_{d},$$

where $r_{d}$ is the dust particle radius, *m* is the complex refractive index of dust particles, and $Q_{sca}$ and $Q_{abs}$ are the scattering and absorption coefficients respectively, which can be obtained as follows

(10) $$Q_{sca}\left(r_{d},\lambda ,m\right)=\frac{2}{d_{s}^{2}}\sum_{n=1}^{\infty }\left(2n+1\right)\left(|a_{n}{|}^{2}+{\left|b_{n}\right|}^{2}\right),$$

(11) $$Q_{abs}\left(r_{d},\lambda ,m\right)=\frac{2}{d_{s}^{2}}\left[\sum_{n=1}^{\infty }\left(2n+1\right)\left(a_{n}+b_{n}\right)-\sum_{n=1}^{\infty }\left(2n+1\right)\left(|a_{n}{|}^{2}+{\left|b_{n}\right|}^{2}\right)\right],$$

where $d_{s}=2\pi r_{d}/\lambda$. Furthermore, $a_{n}$ and $b_{n}$ can be expressed as

(12) $$a_{n}=\frac{\left[\psi_{n}\left(md_{s}\right)\psi_{n}\left(d_{s}\right)\right]\left(m-1\right)}{\left[\psi_{n}\left(md_{s}\right)\xi_{n}\left(d_{s}\right)\right]\left(m-1\right)},$$

(13) $$b_{n}=\frac{\left[\psi_{n}\left(md_{s}\right)\psi_{n}\left(d_{s}\right)\right]\left(1-m\right)}{\left[\psi_{n}\left(md_{s}\right)\xi_{n}\left(d_{s}\right)\right]\left(1-m\right)},$$

where $\psi_{n}\left(d_{s}\right)=d_{s}j_{n}\left(d_{s}\right)$ and $\xi_{n}\left(d_{s}\right)=d_{s}h_{n}^{\left(1\right)}\left(d_{s}\right)$ with $j_{n}\left(d_{s}\right)$ and $h_{n}^{\left(1\right)}\left(d_{s}\right)$ as the spherical Bessel function and spherical first kind Hankel function, respectively.

### 3.2. Analysis of the Interaction of Incident Light with Suspended Dust Particles

**Figure 2:**
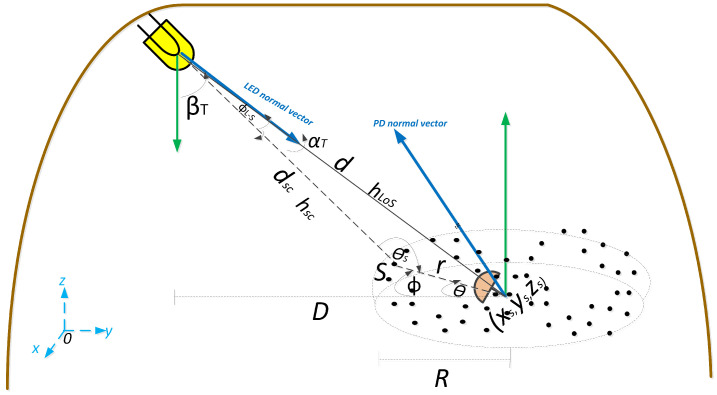
Proposed hemispherical scatterers spatial distribution around the PD for the downlink Cartesian/spherical coordinates relating the LED, an arbitrary dust particle, and the PD.

In UM tunnels, when a light beam interacts with an air molecule (Rayleigh scattering) or a suspended dust particle (Mie scattering), the path that this light beam follows until it reaches the optical receiver is modified. The variable that describes this change is the deflection angle $\theta_{s}$, as can be seen in the geometric pattern of the scattering effect in Figure 2. $\theta_{s}$ is determined by the angular distribution of scattering, namely the scattering phase function $p(\theta_{s})$. Specifically, the generalized Rayleigh scattering phase function is expressed as

(14) $$p_{Ray}\left(\theta_{s}\right)=\frac{3\left[1+3\gamma +\left(1-\gamma \right)\cos^{2}\left(\theta_{s}\right)\right]}{16\pi (1+2\gamma )},$$

where γ=δ/(2−δ) is an atmospheric parameter. On the other hand, the Mie scattering phase function can be represented by the generalized Henyey-Greenstein function as follows:

(15) $$p_{Mie}\left(\theta_{s}\right)=\frac{1-g^{2}}{4\pi }\left[\frac{1}{\sqrt{{\left(1+g^{2}-2g\cos\left(\theta_{s}\right)\right)}^{3}}}+\frac{g(3\cos^{2}\left(\theta_{s}\right)-1)}{2\sqrt{{\left(1+g^{2}\right)}^{3}}}\right],$$

where *g* is an asymmetric atmospheric factor expressed as

(16) $$g=\frac{\int_{0}^{\infty }G\left(r_{d},\lambda ,m\right)n\left(r_{d}\right)dr_{d}}{\int_{0}^{\infty }n\left(r\right)dr_{d}},$$

and *G* is obtained by

(17) $$G\left(r_{d},\lambda ,m\right)=\frac{4}{d_{s}^{2}Q_{sca}}\sum_{n=1}^{\infty }\left[\frac{n(n+2)}{n+1}\left(a_{n}a_{n+1}+b_{n}b_{n+1}\right)+\frac{2n+1}{n(n+1)}\left(a_{n}b_{n}\right)\right].$$

Finally, to consider the entire effect of the interaction of the light beam with dust particles and air molecules, the joint effect of Rayleigh and Mie scattering must be modeled as a Bernoulli distribution. Therefore, the total scattering phase function $p_{total}\left(\theta_{s}\right)$ along with its respective Probability Distribution Function (PDF) are expressed as

(18) $$p_{total}\left(\theta_{s}\right)=\frac{k_{sca}^{Ray}}{k_{sca}}p_{Ray}\left(\theta_{s}\right)+\frac{k_{sca}^{Mie}}{k_{sca}}p_{Mie}\left(\theta_{s}\right),$$

(19) $$f_{sca}\left(\theta_{s}\right)=p_{total}\left(\theta_{s}\right)\sin\left(\theta_{s}\right).$$

### 3.3. The 3D Dust Particles Distribution Model

In this subsection, we present the 3D dust particles distribution model. Figure 2 shows the three-dimensional geometric outline of the UM scenario that relates the LED, the distribution of the dust particles (scatterers) around the receiver, and the PD. Although only one scatter is shown in the Figure 2 for better understanding, there are *N* dust particles around the receiver. Consequently, the expressions obtained are applied to the total number of scatterers that make up the distribution. In this context, for a better description of the scenario, the following preliminary and general assumptions about dust particles were considered:

1. An optical link (transmission path) from the LED to the PD interacts with a single dust particle, being considered an SB scattering model.
2. Suspended dust particles in the UM environment are spherical and uniformly distributed in the mining tunnel, which theoretically tends to infinite.
3. Dust particles behave as reflective isotropic scatterers with similar scatterer coefficients and uniform random phases.

Based on the features, behavior, and distribution of dust particles in UM tunnels and as a baseline to establish a scattering component included in the UM-VLC channel model, we present a random and independently distributed scatterer distribution within a hemispheric 3D region. This distribution is spatially located around the helmet of the mining worker where the PD is located, which is the central point of the hemispheric area as shown in Figure 2.

For simplicity and mathematical convenience, the spatial distribution function of the hemispheroid will be derived considering initially, a uniform general spherical probability distribution/density function, which is expressed as follows

(20) $$f_{x,y,z}^{\left(sp\right)}\left(x_{s},y_{s},z_{s}\right)=\frac{\left(a+1\right)r^{a}}{4\pi R^{\left(a+1\right)}},$$

where *R* is the sphere radius, *r* is the distance from the scatterer *s* to the PD, and a≥0 is the shape factor. In our case, to obtain a spherical 3D shape, we consider a value of a=2. Therefore expression (20) is reformulated as follows

(21) $$f_{x,y,z}^{\left(sp\right)}\left(x_{s},y_{s},z_{s}\right)=\frac{3r^{2}}{4\pi R^{3}}.$$

It is important to mention that outside the spherical region where the scatterers are distributed $f_{x,y,z}^{\left(sp\right)}=0$. The distribution denoted in expression (21) can be easily transformed to derive the corresponding distribution for the hemispheric case considering the following premises:

- $f_{x,y,z}^{\left(sp\right)}=0$ for all underground azimuth angle (ϕ). Therefore, ϕ∉[0,π].
- $f_{x,y,z}^{\left(sp\right)}/2$ for any distribution parameterized by ϕ. Therefore, ϕ∈[π,2π].

In this context, the PDF of the 3D hemispherical distribution of the dust particles is defined as

(22) $$f_{x,y,z}^{\left(he\right)}\left(x_{s},y_{s},z_{s}\right)=\frac{3r^{2}}{2\pi R^{3}}.$$

### 3.4. Joint Distribution of the Arrival Time and the Arrival Angle of the Scattering Downlink Link

As mentioned above, although there are *N* scatterers uniformly distributed within the hemispheric region, for our analysis, a scatterer (denoted by *s*) is considered in a random position ($x_{s},y_{s},z_{s}$) in the Cartesian coordinates and (*r*, θ, ϕ) in the spherical coordinates, where r≥0 the elevation angle (θ) is defined for 0≤θ≤π and ϕ is defined for π≤ϕ≤2π.

#### 3.4.1. Cartesian-Spherical Coordinates Transformations

As can be seen in Figure 2, an arbitrary scatterer will have spherical coordinates (*r*, θ, ϕ). Therefore, based on the geometric characteristics of the stage and for convenience in handling the notation, the spherical coordinates related to the Cartesian coordinates ($x_{s},y_{s},z_{s}$) are expressed as follows

(23) $$\left(x_{s},y_{s},z_{s}\right)=\left(r\cos\phi ,r\sin\phi \cos\theta ,r\sin\phi \sin\theta \right),$$

(24) $$r=\sqrt{x_{s}^{2}+y_{s}^{2}+z_{s}^{2}},$$

(25) $$\theta =\arctan\left(\frac{z_{s}}{y_{s}}\right),$$

(26) $$\phi =\arccos\left(\frac{x_{s}}{r}\right)=\arccos\left(\frac{x_{s}}{\sqrt{x_{s}^{2}+y_{s}^{2}+z_{s}^{2}}}\right).$$

Furthermore, based on the premise that any Cartesian-defined PDF function can be expressed in terms of spherical coordinates, we can restructure the $f_{x,y,z}^{\left(he\right)}\left(x_{s},y_{s},z_{s}\right)$ expression as follows

(27) $$f_{r,\theta ,\phi }^{\left(he\right)}\left(r,\theta ,\phi \right)=\frac{f_{x,y,z}^{\left(he\right)}\left(x_{s},y_{s},z_{s}\right)}{\left|J(x_{s},y_{s},z_{s})\right|}{|}_{\begin{array}{cc} x_{s}=r\cos\phi & \\ y_{s}=r\sin\phi \cos\theta & \\ z_{s}=r\sin\phi \sin\theta \end{array}},$$

where $J(x_{s},y_{s},z_{s})$ is the Jacobian transformation given by

(28) $$J\left(x_{s},y_{s},z_{s}\right){|}_{\begin{array}{cc} x_{s}=r\cos\phi & \\ y_{s}=r\sin\phi \cos\theta & \\ z_{s}=r\sin\phi \sin\theta \end{array}}={\left|\begin{array}{ccc} \frac{\delta x_{s}}{\delta r} & \frac{\delta x_{s}}{\delta \theta } & \frac{\delta x_{s}}{\delta \phi }\\ \frac{\delta y_{s}}{\delta r} & \frac{\delta y_{s}}{\delta \theta } & \frac{\delta y_{s}}{\delta \phi }\\ \frac{\delta z_{s}}{\delta r} & \frac{\delta z_{s}}{\delta \theta } & \frac{\delta z_{s}}{\delta \phi } \end{array}\right|}^{-1}=-{\left(r^{2}\sin\phi \right)}^{-1}.$$

Hence, by evaluating expression (27) and including expression (28) in it, we obtain the expression of $f_{r,\theta ,\phi }^{\left(he\right)}$ independent of θ as follows

(29) $$f_{r,\theta ,\phi }^{\left(he\right)}\left(r,\theta ,\phi \right)=r^{2}\sin\phi f_{x,y,z}^{\left(he\right)}\left(x_{s},y_{s},z_{s}\right)=r^{2}\sin\phi f_{x,y,z}^{\left(he\right)}\left(r\cos\phi ,r\sin\phi \cos\theta ,r\sin\phi \sin\theta \right).$$

#### 3.4.2. Derivation of the Arrival Time and Arrival Angle Joint PDF

By defining τ as the arrival time of the signal that travels from the LED to the PD, The total length of the optical communication path from the LED to the dust particle and then to the PD is given by

(30) $$r+d_{sc}=c\tau ,$$

where $d_{sc}$ is the distance between scatterer *S* and the LED and *c* is the speed of light. By using consine law and trigonometric equations, *r* can be given as

(31) $$d_{sc}=\sqrt{D^{2}+r^{2}-2Dr\sin\theta \cos\phi },$$

where *D* is the separation distance between the LED and the PD on the y-axis. Hence, *r* can be obtained from (30) and (31) as

(32) $$r=\frac{{\left(c\tau \right)}^{2}-D^{2}}{2\left(c\tau -D\sin\theta \cos\phi \right)},$$

The variable τ can be incluided in the expression (27) in order to determinate the arrival time and arrival angle joint PDF, $f_{\tau ,\theta ,\phi }^{\left(he\right)}\left(\tau ,\theta ,\phi \right)$, which is expressed as

(33) $$f_{\tau ,\theta ,\phi }^{\left(he\right)}\left(\tau ,\theta ,\phi \right)=\frac{r^{2}\sin\phi f_{x,y,z}^{\left(he\right)}\left(x_{s},y_{s},z_{s}\right)}{\left|J(r,\theta ,\phi )\right|}{|}_{\begin{array}{c} r=\frac{{\left(c\tau \right)}^{2}-D^{2}}{2\left(c\tau -D\sin\theta \cos\phi \right)} \end{array}}.$$

where the Jacobian transformation J(r,θ,ϕ) is given by

(34) $$J\left(r,\theta ,\phi \right)=|\frac{\delta r}{\delta \tau }{|}^{-1}=\frac{2{\left(c\tau +D\sin\theta \cos\phi \right)}^{2}}{c\left[{\left(c\tau \right)}^{2}+2c\tau D\sin\theta \cos\phi +D^{2}\right]}.$$

Substituting (34) into (33), we can obtain $f_{\tau ,\theta ,\phi }^{\left(he\right)}\left(\tau ,\theta ,\phi \right)$ as

(35) $$f_{\tau ,\theta ,\phi }^{\left(he\right)}\left(\tau ,\theta ,\phi \right)=\frac{c{\left[{\left(c\tau \right)}^{2}-D^{2}\right]}^{2}\left[{\left(c\tau \right)}^{2}+2c\tau D\sin\theta \cos\phi +D^{2}\right]\sin\phi }{8{\left(c\tau +D\sin\theta \cos\phi \right)}^{4}}f_{x,y,z}^{\left(he\right)}\left(x_{s},y_{s},z_{s}\right).$$

Finally, by replacing the expression of *r* (Equation (32)) in (21) and including this result within (35), we can obtain the joint PDF of the arrival time and the arrival angle only in terms of the variables of interest τ, θ, and ϕ as follows

(36) $$f_{\tau ,\theta ,\phi }^{\left(he\right)}\left(\tau ,\theta ,\phi \right)=\frac{c{\left[{\left(c\tau \right)}^{2}-D^{2}\right]}^{2}\left[{\left(c\tau \right)}^{2}+2c\tau D\sin\theta \cos\phi +D^{2}\right]\sin\phi }{8{\left(c\tau +D\sin\theta \cos\phi \right)}^{4}}\times \frac{3r^{2}}{2\pi R^{3}},$$

(37) $$f_{\tau ,\theta ,\phi }^{\left(he\right)}\left(\tau ,\theta ,\phi \right)=\frac{3c{\left[{\left(c\tau \right)}^{2}-D^{2}\right]}^{4}\left[{\left(c\tau \right)}^{2}+2c\tau D\sin\theta \cos\phi +D^{2}\right]\sin\phi }{64\pi {\left(c\tau +D\sin\theta \cos\phi \right)}^{4}{\left(c\tau -D\sin\theta \cos\phi \right)}^{2}R^{3}}.$$

### 3.5. UM-VLC Channel Component Produced by Scattering

We assume that each scatterer *s* introduces a coefficient *G*. This coefficient can be determined mathematically as $G=\bar{\rho }\left(\lambda \right)/N$[12]. However, since the interaction of light with dust particles is probabilistic, it is necessary to include in this gain the PDF of the total scattering phase function. Therefore, *G* is redefined as

(38) $$G\left(\theta_{s}\right)=\frac{\bar{\rho }\left(\lambda \right)f_{sca}\left(\theta_{s}\right)}{N}.$$

Since the interaction of light with dust particles is considered as a VLC channel component produced by the optical signal reflection until it reaches the receiver and based on the typical Lambertian VLC channel modeling, the mathematical expressions for the Direct Current (DC) gain and the CIR of the UM-VLC channel component produced by dust particles can be written as

(39) $$H_{sca}=\lim_{N\to \infty }\sum_{n=1}^{N}\frac{A_{p}\left(m+1\right)G\left(\theta_{s}\right)}{2\pi {\left(r+d_{sc}\right)}^{2}}\cos^{m}\left(\phi_{L-S}\right)\cos\left(\theta \right)rect\left(\frac{\theta }{\Theta }\right),$$

(40) $$h_{sca}\left(t\right)=\lim_{N\to \infty }\sum_{n=1}^{N}\frac{A_{p}\left(m+1\right)G\left(\theta_{s}\right)}{2\pi {\left(r+d_{sc}\right)}^{2}}\cos^{m}\left(\phi_{L-S}\right)\cos\left(\theta \right)rect\left(\frac{\theta }{\Theta }\right)\delta \left(t-\frac{r+d_{sc}}{c}\right),$$

where $A_{p}$ is the PD physical active area, $m=-1/log_{2}\left[\cos(\phi_{1/2})\right]$ represents the Lambertian mode number of the LED, which is a function of semi-angle at half power ($\phi_{1/2}$) of the LED, Θ is the PD Field of View (FoV), $\phi_{L-S}$ is the radiance angle measured between the the normal vector to the LED surface and the vector from the LED to the scatterer *s*, and δ(·) is a unit-area Dirac delta function.

Furthermore, the total power received by the PD due to the luminous intensity emitted by the LED can be described as

(41) $$P_{r}=R_{PD}P_{t}h_{m}+n_{m},$$

where $R_{PD}$ is the PD responsivity, and $P_{t}$ is the emitted optical power by the LED.

Finally, The SNR is computed by using the metric $P_{r}$, whose expression is given as

(42) $$SNR=\frac{P_{r}^{2}}{\sigma_{s}^{2}+\sigma_{t}^{2}},$$

## 4. Results and Discussions

In this section, we simulate, implement, and present theoretical results obtained by computer software in terms of multiple evaluation parameters of wireless communication systems based on the proposed theoretical channel model in order to examine the capacity, behavior, and performance of the UM-VLC system. Furthermore, we make a fair comparison with the UM-VLC channel model and its scenario developed in [12]. Among the parameters that are validated are the CIR, the received power, the RMS delay spread, the SNR, and the BER. Both the parameters of the simulation model that were used in this manuscript as well as the description of the UM scenario are presented in Table 1.

### 4.1. Channel Impulse Response

Using LoS, NLoS, and scattering ray tracing methodology, we determine the detected power and path lengths from the LED to the PD for each ray. Then, these data are processed to produce the UM-VLC CIR curves.

**Figure 3:**
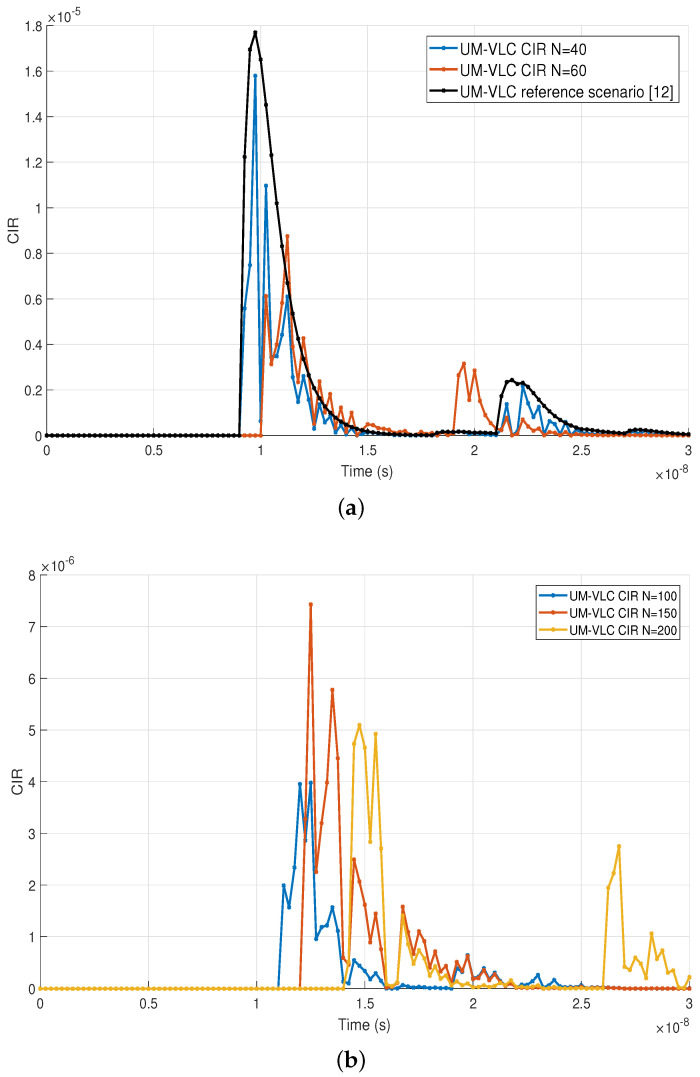
UM-VLC CIR curves for (**a**) 40 and 60 dust particles in the hemispheric area, and UM-VLC reference scenario, Reprinted from Ref. [12], and (**b**) 100, 150, and 200 dust particles in the hemispheric area.

In the context of analyzing the effect on the complete UM-VLC channel model of the number of dust particles in the hemispherical distribution, Figure 3 shows the CIR for different values of *N*. Figure 3a compares the UM-VLC CIRs for values of 40 and 60 dust particles with the UM-VLC reference scenario developed in [12]. We can observe that with the value of 60 dust particles considered in the hemispherical distribution, there is a decrease in the magnitude of the UM-VLC CIR and a greater temporal dispersion of its components compared to the curve for *N* = 40. If we compare both curves with the UM-VLC reference scenario, we notice a decrease in the magnitude of the CIRs due to the more precise modeling of the scattering that is achieved with the hemispherical distribution of dust particles. On the other hand, Figure 3b compares the UM-VLC CIRs for *N* values of 100, 150, and 200 dust particles. We can notice that for the values of *N* = 150 and *N* = 200 the maximum values of the UM-CIR are similar. Therefore, it could be considered that for values greater than 150 dust particles, the UM-VLC CIR has similar behavior. This is because the scattering component is not the only factor that is influencing the CIR. The full UM-VLC channel model is influenced by random LED and PD orientations, non-regular walls, and shadowing. As a general finding, it could be considered that when we increase the value of *N*, the light scattering affects the LoS component to a greater extent, in terms of magnitude and time.

**Figure 4:**
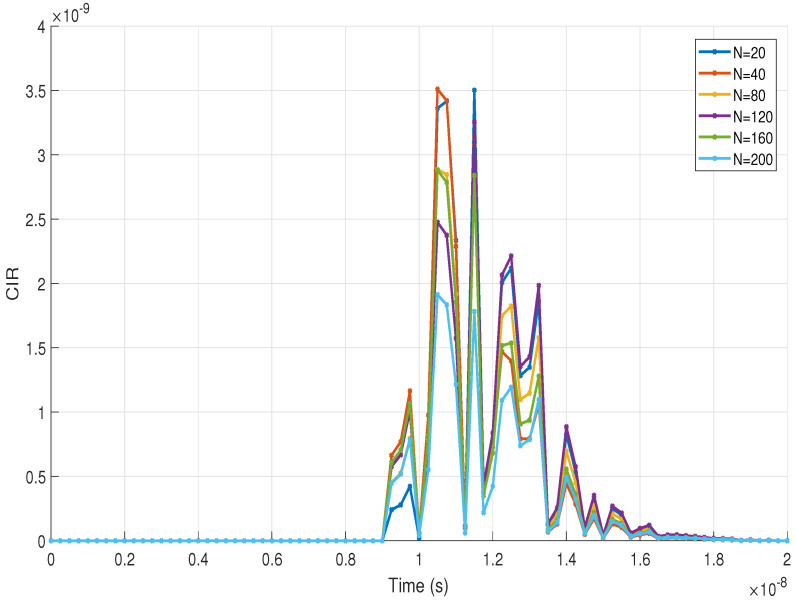
CIR of the scattering component with different values of *N* in the evaluated UM scenario.

The specific approach and analysis of the scattering component based on the variation of *N* values are presented in Figure 4. Here, *N* was varied between values of 20 and 200 dust particles in order to observe their behavior and differences in the CIR curves. It is possible to notice that for all the values of *N* chosen, the temporal behavior of the CIR curves is the same. Therefore, we can denote that the hemispherical distribution has the same behavior regardless of the value of *N*. However, and confirming the finding in Figure 3, we can observe that for values of *N* greater than 120, the CIR curves have similar magnitudes, which vary between them by a factor of $0.2\times 10^{-9}$. For a better comparative interpretation of the CIRs curves shown in Figure 4, we can verify the maximum magnitude values of the CIRs by varying *N* in Table 2.

### 4.2. Received Power

**Figure 5:**
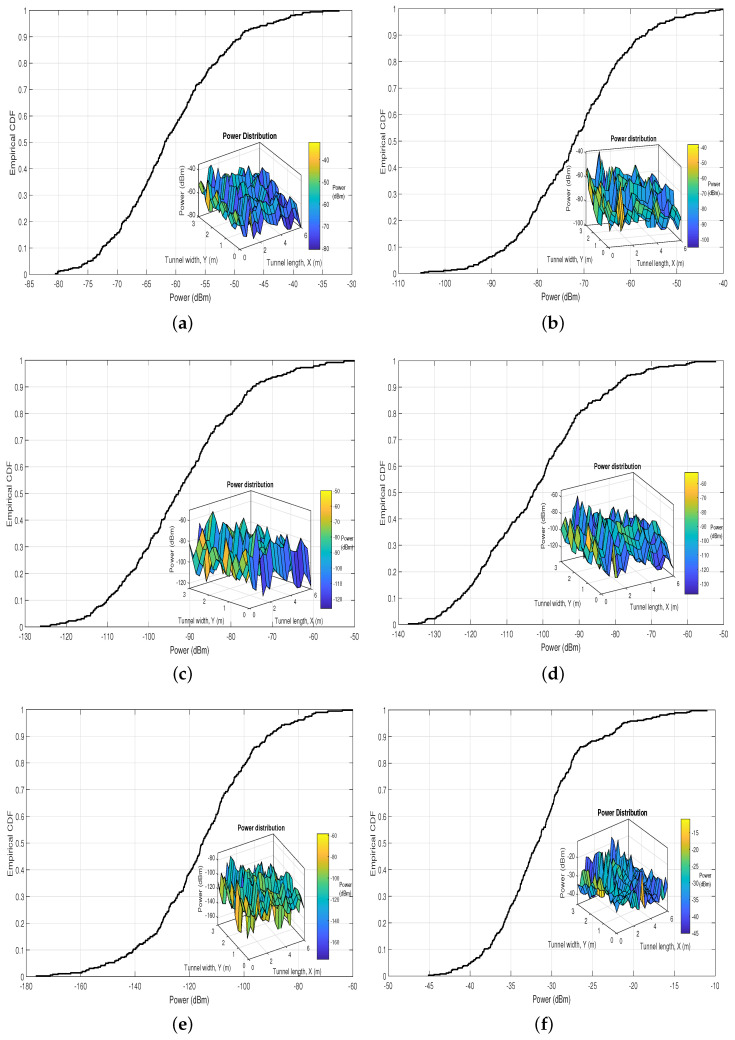
Empirical CDF and distribution of the received power in the UM-VLC scenario with (**a**) 40, (**b**) 60, (**c**) 100, (**d**) 150, (**e**) 200 dust particles in the hemispheric area, and (**f**) UM-VLC reference scenario, Reprinted from Ref. [12].

Figure 5 shows the empirical Cumulative Distribution Functions (CDFs) of the power received by the PD in the UM scenario for values of *N* between 40 and 200, which is computed based on the Equation (41). Furthermore, in each graph, we insert the power received distribution in the UM scenario. We evaluate the received power by keeping the *z* coordinate of the PD with a value of 1.8 m because it is a typical position value of communication devices in these scenarios. Also, $P_{t}$ is set to 5 W, to provide constant illumination in the tunnel.

Compared with the CDF and distribution of the received power obtained in the work presented in [12] (see Figure 5f), the maximum and minimum values of the received power obtained in this study for all the values of *N* are smaller. This finding implies that the derived hemispherical dust particle distribution model presents a greater similarity with a real UM scenario since the negative effect of scattering on the received power is more noticeable. In fact, if we compare the CDFs and distributions of received power for the chosen values of *N*, we can notice that if we increase the value of *N*, the effect on the decrease of the received power is greater, even though the power distribution remains quite irregular for all values of *N*.

### 4.3. Signal to Noise Ratio

**Figure 6:**
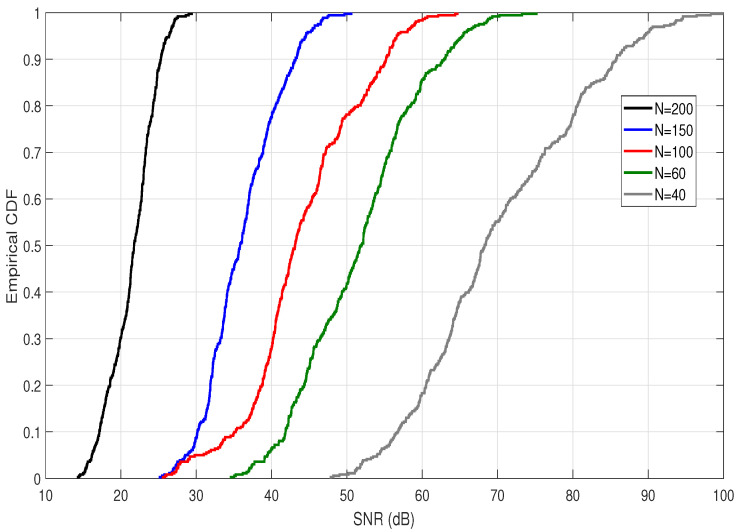
Empirical CDF of the SNR obtained for different values of *N* in the hemispheric area of the UM-VLC scenario evaluated.

The complete UM-VLC model and the effect on it of the scattering channel component together with the presented hemispherical distribution is also evaluated by implementing the SNR metric, which is calculated based on Equation (42) and presented as empirical CDF curves. As we can see in the expression (42), the received power is used to compute the SNR. Therefore, the dependence of the SNR on the received power and its trend is reflected in the CDFs curves obtained. In this context, Figure 6 shows the empirical CDFs of the SNR for different *N* values between 40 and 200 dust particles.

Based on these curves, several important findings are highlighted. We observe that the best SNR performance of the UM-VLC system is obtained when *N* = 40 and the worst performance is obtained when *N* = 200. This reaffirms the idea based on the results of the previous subsections, which implies that as the value of *N* increases, the performance of the UM-VLC system decreases. However, for values of *N* = 100 and *N* = 150 and for an SNR between 27 and 29 dB, the CDF curves have similar behavior. This behavior allows us to infer that, between these SNR values, the effect of increasing dust particles is not decisive. We must also remember that not only scattering channel component is evaluated, but we also evaluate the UM-VLC channel with all the environmental factors that affect it.

### 4.4. Delay Spread

The RMS delay spread is a fundamental metric in wireless communications, which allows us to measure the effect of multi-path propagation in this type of system. In our case, it will allow us to evaluate the multi-path effect of the UM-VLC channel components in the UM environment since this model has components that generate temporal dispersion, which degrades the channel bandwidth. The irregular tunnel walls and dust particles in the environment generate multiple random reflections and scattering of the original light signal. These effects cause splitting of the signal that propagates through the channel in different paths and distances, which arrive at the PD in a different period of time and induce a delay. The time difference between the arrival of signals at the PD and the particular delay after the arrival of the first light signal, which is usually the LoS component, is declared as excess delay $\tau_{p}$.

The temporal dispersion has a direct relation with the UM-VLC CIR. Therefore, the RMS delay spread of the UM-VLC channel can be expressed as

(43) $$D_{RMS}=\sqrt{\frac{\sum_{p=0}^{P}{\left(\tau_{p}-\mu_{RMS}\right)}^{2}\;{h_{m}^{2}}_{p}}{\sum_{p=0}^{P}{h_{m}^{2}}_{p}}},$$

where ${h_{m}^{2}}_{p}$ represents the sum of samples numeric of all the channel components that form it with a maximum number of samples *P* and the mean delay spread $\mu_{RMS}$, which is given by [16]

(44) $$\mu_{RMS}=\frac{\sum_{p=0}^{P}\tau_{p}\;{h_{m}^{2}}_{p}}{\sum_{p=0}^{P}{h_{m}^{2}}_{p}}.$$

**Figure 7:**
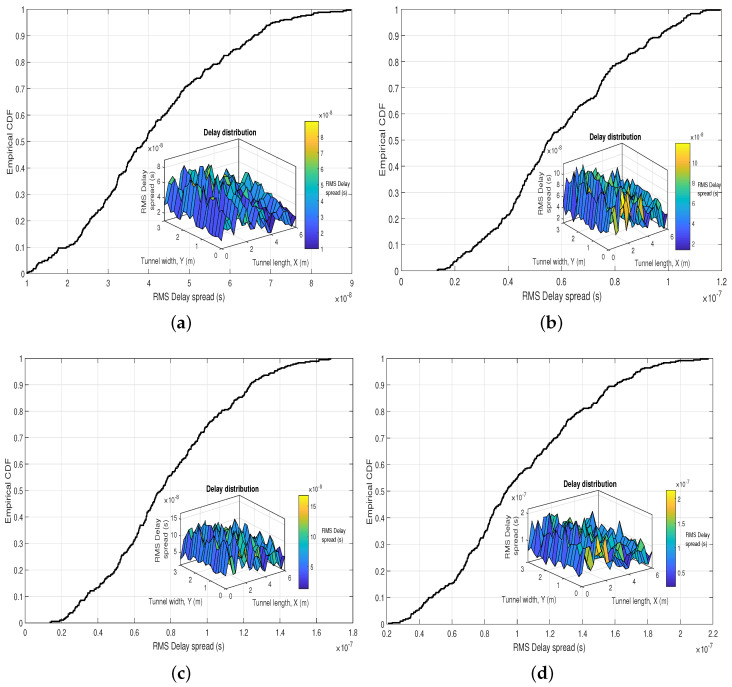

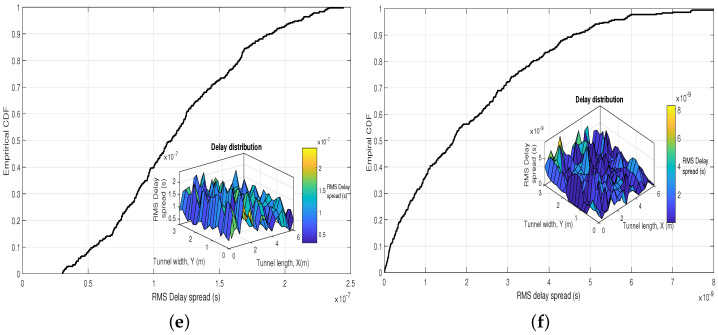
Empirical CDF and distribution of the RMS Delay spread in the UM-VLC scenario with (**a**) 40, (**b**) 60, (**c**) 100, (**d**) 150, (**e**) 200 dust particles in the hemispheric area, and (**f**) UM-VLC reference scenario, Reprinted from Ref. [12].

The behavior of the RMS delay spread metric is plotted in terms of empirical CDFs and distributions in the UM-VLC scenario by varying the *N* values between 40 and 200 dust particles, as can be seen in Figure 7. If we compare the result of RMS delay spread obtained in work [12] (see Figure 7f), with any of the curves and distributions obtained in Figure 7, we can notice that the numerical values obtained in this manuscript for any value of *N* are higher. This finding would also contribute to the deduction that the model of hemispherical dust particles presented in this work is closer to the reality of the UM-VLC scenario because the light signal has a more evident time delay. Although all RMS delay spread distributions for all *N* values share the same random trend in the UM scenario, we can observe that the higher the *N* values, the RMS delay spread values also tend to grow.

### 4.5. Bit Error Rate

A very important metric that allows us to evaluate the performance of the complete UM-VLC system is the BER. By using this metric we can find the number of bits that have been altered by the effects of the mining environment, including the effect of scattering by dust particles. This evaluation is carried out using Monte Carlo simulations and integrating the features of the UM tunnel and the UM-VLC channel model. the LED transmission is based on the PHY-I mode together with the On-Off Keying (OOK) modulation, which are described in the IEEE 802.1.5.7 standard. On the other hand, the PD reception employs asynchronous symbol-by-symbol detection [25,26].

**Figure 8:**
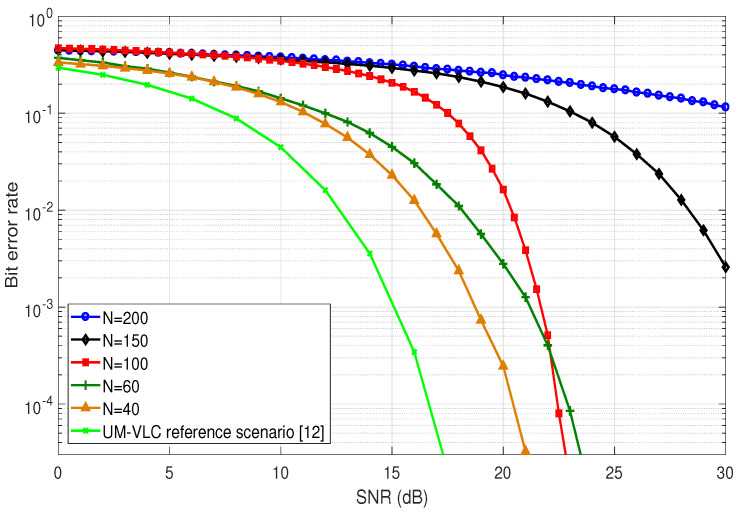
BER curves for different values of *N* in the hemispheric area of the evaluated UM-VLC scenario and the UM-VLC reference scenario, Reprinted from Ref. [12].

Figure 8 shows the BER curves obtained for different values of *N* applied to the hemispherical distribution of dust particles in the UM-VLC scenario and the UM-VLC reference scenario [12]. It is important to emphasize that the curve obtained from the UM-VLC reference scenario is the one that presents the best performance, due to the fact that the implemented scattering model is basic and unrealistic (2D disk-shaped distribution model) [12]. However, if we compare the curves determined with the UM-VLC model using the hemispherical distribution of dust particles, we can clearly see in Figure 8 that the BER curve with the best performance is the one that implements a value of *N* = 40. This result was quite expected by all the factors analyzed in the previous subsections. On the other hand, for values of *N* = 150 and *N* = 200, the system presents the worst performance in terms of BER. This result allows to reinforces the criterion of the dependence of the UM-VLC system performance in terms of the number of dust particles that exist in the proposed hemispherical distribution model. Finally, it was verified that the performance of the UM-VLC system not only depends on the scattering phenomenon, but also on the other physical variables proper to the general UM-VLC channel model.

## 5. Conclusions

In this paper, we present an analysis of a SISO UM-VLC system with dust particles suspended in the UM environment and atmospheric parameters. Furthermore, and as the main contribution of this manuscript, we propose a hemispherical 3D dust particle distribution model that more accurately represents the UM hostile environment. The mathematical modeling and the derivation of the statistical parameters of this scatter distribution model allows to present a more coherent scattering channel component produced by the dust particles and to establish a precise and compact general mathematical model of the UM-VLC channel. The performance of the dust particle distribution analytical framework and the improvement of the UM-VLC channel model are evaluated and verified in a UM-VLC system based on computational numerical simulations based on the IEEE 802.1.5.7 standard by varying the number of distributed dust particles in terms of CIR, received power, SNR, RMS delay spread, and BER. The findings based on the CIR are relevant to verify that the temporal dispersion for any number of dust particles is higher compared to similar works in the literature. However, when comparing the curves with values of *N* = 150 and *N* = 200, we observe that they are very similar (approximately between 4 × $10^{-6}$ and 5 × $10^{-6}$ at their maximum values). These results demonstrate that as the amount of suspended dust particles increases, the CIR decreases in magnitude. The results of the received power, SNR, and RMS delay spread are presented as empirical CDFs and distributions in the UM scenario by varying the number of dust particles. These findings reaffirm that the 3D hemispherical dust particle distribution model derived in this article describes more accurately the UM scenario with respect to basic dust particle distributions found in the literature. This is due to the fact that the real interaction of the light with the scatterers, in general, worsens the performance of the UM-VLC system since the power received and the SNR decrease, and greater time delays are generated. Finally, the system has a better performance in terms of BER when fewer dust particles are considered. On the other hand, the worst performance of the curves occurs for values of *N* = 150 and *N* = 200. These findings reinforce the criteria that the system performance depends directly on the number of dust particles found in the distribution implemented in the UM-VLC scenario.

By presenting a more accurate UM-VLC channel model through the enhancement of the channel component produced by scattering, as future work, it is necessary to present solutions that mitigate the problems generated in the UM-VLC system when there is a greater amount of dust particles. Among these solutions are reception mechanisms based on machine learning and angular diversity receivers. Furthermore, we will validate experimentally the UM-VLC channel model including the scattering components and the distribution of dust particles proposed in this article. This experimental test-bed will have all the features of a UM tunnel to verify the behavior of the VLC system in this environment. Furthermore, and as future work, a deeper statistical development (second-order statistics and probability distributions) of the hemispherical distribution of dust particles, the behavior of dust particles when interacting with light, and the UM-VLC scattering channel model component should be evaluated and analyzed.

## Figures and Tables

**Table 1:** UM-VLC system simulation parameters.

UM Simulation Scenario	Values	References
**Tunnel Parameters**		
Dimensions (w×l×h)	(3×6×5) m	
Coordinates of the LED(x,y,z)	(0.5×3×4.5) m	
Coordinates of the PD(x,y,z)	(1×3×1.8) m	
**Channel parameters**		
Absorption coefficient, ($Q_{abs}$)	1.3	[23]
Atmospheric parameter, γ	0.017	[12]
Atmospheric parameter, *g*	0.72	[12]
Atmospheric parameter, *f*	0.5	[12]
AWGN power spectral density (A/Hz)	$2.5\times 10^{-23}$	[28]
LED rotation angle, $\alpha_{i}\;{(}^{\circ })$	45	[26]
LED tilt angle, $\beta_{i}\;{(}^{\circ })$	45	[26]
Noise bandwidth (MHz)	100	[29]
PD rotation angle, $\alpha_{i}\;{(}^{\circ })$	45	[26]
PD tilt angle, $\beta_{i}\;{(}^{\circ })$	45	[26]
Scatterer reflection coefficient, $\rho_{s}$	0.1	[23]
Scattering coefficient, ($Q_{sca}$)	0.4	[23]
Sphere radius, *R* (m)	1	[16,20]
Wall reflection coefficient, $\rho_{w}$	0.6	[26]
Wall rotation angle, $\alpha_{w}\;{(}^{\circ })$	*U*[0,180]	[26]
Wall tilt angle, $\beta_{w}\;{(}^{\circ })$	*U*[0,180]	[26]
**VLC transceiver parameters**		
Average transmitted power, $P_{t}$ (W)	5	[23]
Band-pass filter of transmission	1	[6]
Dust concentration *C* ($\mathrm{mg}/m^{3}$)	15	[18,19]
Dust particle radius, $r_{d}$ (μm)	*U*[0.2,2]	[18,19]
FoV, $\Theta \;{(}^{\circ })$	70	[23]
Gain of the optical filter	1	[23]
Lambertian mode number, *m*	1	[16,20]
LED wavelength, λ (nm)	580	[23]
Modulation type	OOK	[9]
Modulation bandwidth (MHz)	50	[9]
Modulation index	0.3	[9]
Optical filter bandwidth (nm)	340 to 694.3	[23]
Optical filter center wavelength (nm)	580 ± 2	[23]
Optical filter full width half max (nm)	10 ± 2	[23]
Physical active area, $A_{p}$ (cm${}^{2}$)	1	[6]
Reflective element area, $\Delta A_{w}\;\left({\mathrm{cm}}^{2}\right)$	1	[12]
Refractive index, $m_{s}$	1.5	[6]
Responsivity, $R_{PD}$ (A/W)	0.53	[6]
Semi-angle at half power, $\phi_{i1/2}\;{(}^{\circ })$	60	[23]

**Table 2:** Maximum CIR values for each value of *N*.

*N*	CIR Maximum Value
20	$3.53\times 10^{-9}$
40	$3.45\times 10^{-9}$
80	$3.18\times 10^{-9}$
120	$2.83\times 10^{-9}$
160	$2.55\times 10^{-9}$
200	$2.23\times 10^{-9}$

## Data Availability

The data presented in this study are available on request from the corresponding author.

## Abbreviations

The following abbreviations are used in this manuscript:

AWGN	Additive White Gaussian Noise
BER	Bit Error Rate
CDF	Cumulative Distribution Function
CIR	Channel Impulse Response
DC	Direct Current
FoV	Field-of-View
GDP	Gross Domestic Product
LED	Light-Emitting Diode
LoS	Line-of-Sight
MISO	Multiple-Input Single-Output
MODTRAN	Atmospheric Radiation Transport Model
NLoS	Non-Line-of-Sight
OOK	On-Off Keying
PD	Photo-Diode
PDF	Probability Distribution Function
RS-GBSM	Regular-Shaped Geometry-Based Stochastic Model
RMS	Root Mean Square
UM	Underground Mining
SB	Single-Bounced
SISO	Single-Input Single-Output
SNR	Signal-to-Noise-Ratio
VLC	Visible Light Communication
